# Lung MRI with hyperpolarised gases: current & future clinical perspectives

**DOI:** 10.1259/bjr.20210207

**Published:** 2021-06-09

**Authors:** Neil J Stewart, Laurie J Smith, Ho-Fung Chan, James A Eaden, Smitha Rajaram, Andrew J Swift, Nicholas D Weatherley, Alberto Biancardi, Guilhem J Collier, David Hughes, Gill Klafkowski, Christopher S Johns, Noreen West, Kelechi Ugonna, Stephen M Bianchi, Rod Lawson, Ian Sabroe, Helen Marshall, Jim M Wild

**Affiliations:** 1 POLARIS, Department of Infection, Immunity & Cardiovascular Disease, University of Sheffield, Sheffield, UK; 2 Sheffield Children's NHS Foundation Trust, Sheffield, UK; 3 Directorate of Respiratory Medicine, Sheffield Teaching Hospitals NHS Trust, Sheffield, UK; 4 Insigneo Institute of In Silico Medicine, Sheffield, UK

## Abstract

The use of pulmonary MRI in a clinical setting has historically been limited. Whilst CT remains the gold-standard for *structural* lung imaging in many clinical indications, technical developments in ultrashort and zero echo time MRI techniques are beginning to help realise non-ionising *structural* imaging in certain lung disorders. In this invited review, we discuss a complementary technique – hyperpolarised (HP) gas MRI with inhaled ^3^He and ^129^Xe – a method for *functional* and *microstructural* imaging of the lung that has great potential as a clinical tool for early detection and improved understanding of pathophysiology in many lung diseases. HP gas MRI now has the potential to make an impact on clinical management by enabling safe, sensitive monitoring of disease progression and response to therapy. With reference to the significant evidence base gathered over the last two decades, we review HP gas MRI studies in patients with a range of pulmonary disorders, including COPD/emphysema, asthma, cystic fibrosis, and interstitial lung disease. We provide several examples of our experience in Sheffield of using these techniques in a diagnostic clinical setting in challenging adult and paediatric lung diseases.

## Introduction

Pulmonary MRI has historically had limited clinical impact due to the poor image signal-to-noise and short T2* caused by magnetic susceptibility differences between air and lung parenchyma. Whilst CT remains the gold-standard for structural lung imaging in many clinical indications, the advent of ultrashort and zero echo time (UTE/ZTE) acquisition techniques has enabled ^1^H pulmonary MRI to advance to a point such that it is now recommended clinically for certain disorders.^1^ Hyperpolarised (HP) gases, ^3^He and ^129^Xe, as inhaled MRI contrast agents offer a wealth of complementary information about the function and microstructure of the lung and have great potential as a clinical tool for early detection and understanding of patho-physiology of certain lung diseases. HP gas MRI is now poised to impact upon clinical management through safe, sensitive monitoring of disease progression and response to therapy.

In this invited review, we briefly introduce HP gas MRI techniques and what aspects of lung function they probe. Then, focusing on the substantial evidence base gathered to date, we review HP gas MRI studies in patients with a range of pulmonary disorders, including emphysema, asthma, cystic fibrosis, and interstitial lung disease. Finally, we review our experience of using the technique in Sheffield in a diagnostic clinical setting.

### HP gas MRI methodology

Gases are intrinsically low spin density and conventional polarisation by the scanner’s magnetic field generally provides a weak signal for imaging, although encouraging results with thermally polarised fluorinated gases are emerging for imaging lung ventilation.^2,3^ However, for certain gases such as ^3^He and ^129^Xe, the MR signal can be boosted by 4—5 orders of magnitude through hyperpolarisation, typically achieved through spin-exchange optical pumping with a high-powered laser.^4^ Once hyperpolarised, these gases can be delivered to a patient in up to 1 L doses via a plastic bag. MR image acquisition is usually performed during a short breath-hold (<15 s) after inhalation from the bag, and throughout which vital signs are monitored.

We now briefly review the different aspects of lung function, physiology and microstructure that the method can be used to investigate.

Ventilation: Direct MR imaging of HP gas in the lungs provides a measure of gas density and allows the visualisation of the distribution and heterogeneity of lung *ventilation*; *i.e*. the delivery of inspired gas to the alveoli and distal airways. Ventilation abnormalities/defects – signal voids in the image – reflect the absence of HP gas in affected regions of the lung and can be caused by obstruction or constriction of the airways.

**Figure 1.**
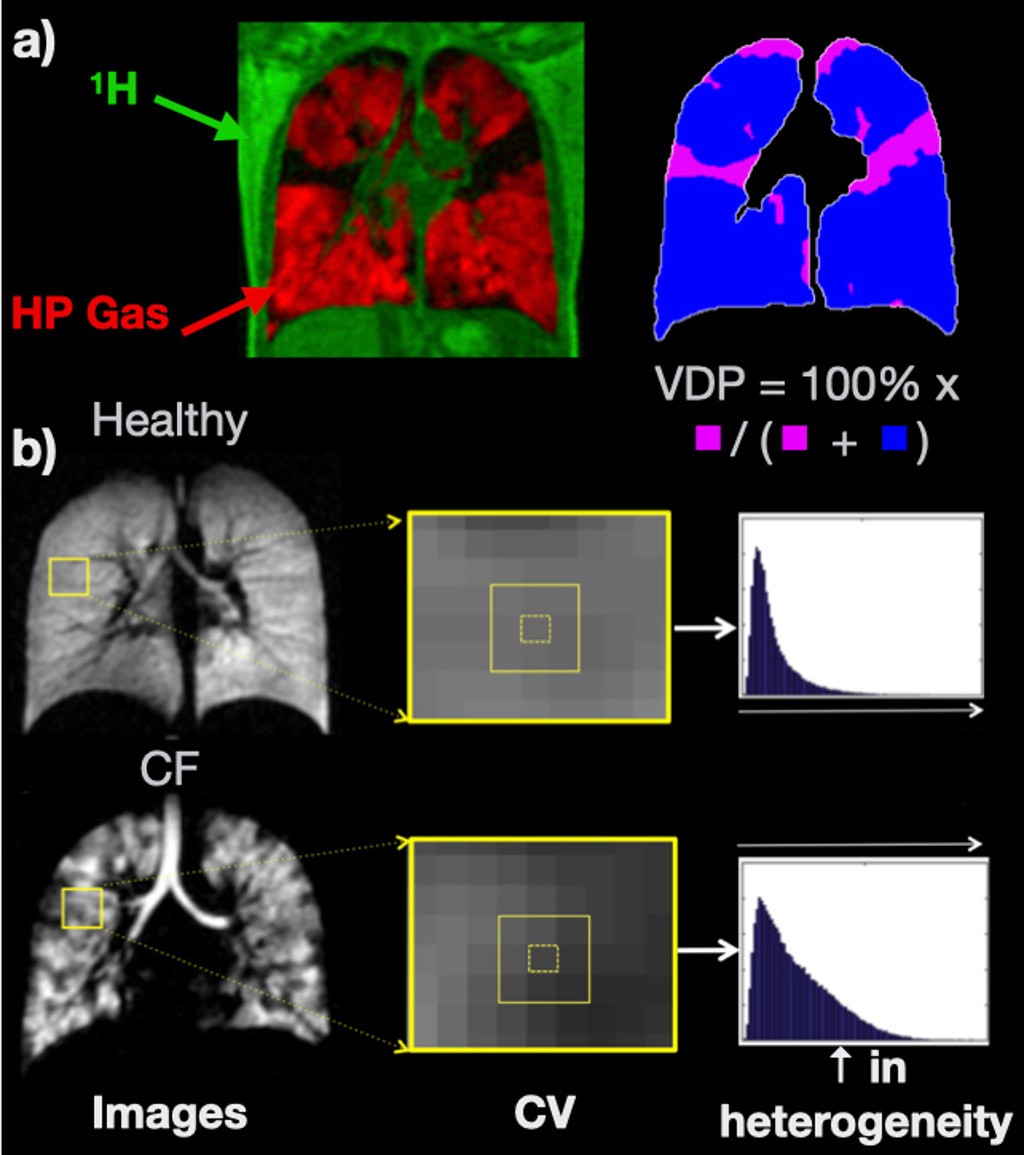
a) Example HP gas ventilation image (red) overlaid on a ^1^H MRI anatomical image (green). VDP is derived by identifying low / no signal voxels (defects; pink) and calculating their volume percentage of the whole of the lungs as determined from ^1^H MRI. (**b**) HP gas ventilation images of the lungs of a healthy child and a child with CF, exhibiting homogeneous and heterogeneous ventilation, respectively. CV is derived by sliding a kernel (in this case 3 × 3) over the image and calculating the coefficient of variation for the central voxel in the window. The mean, median or interquartile range of the resulting CV histogram can be quoted. CF, cystic fibrosis; CV, coefficient of variation; HP, hyperpolarised; VDP, ventilation defect percentage.

Ventilation biomarkers: Ventilation defect percentage (VDP); the percentage of low/zero intensity pixels in the image, or its inverse, the ventilated volume percentage (VV%), are the most-commonly used biomarkers of ventilation (Figure 1a). Ventilation heterogeneity can be further quantified by calculating the coefficient of variation (CV)^5,6^ (Figure 1b), or classifying pixels into defect, low, normal and high ventilation bins.^7^

**Figure 2.**
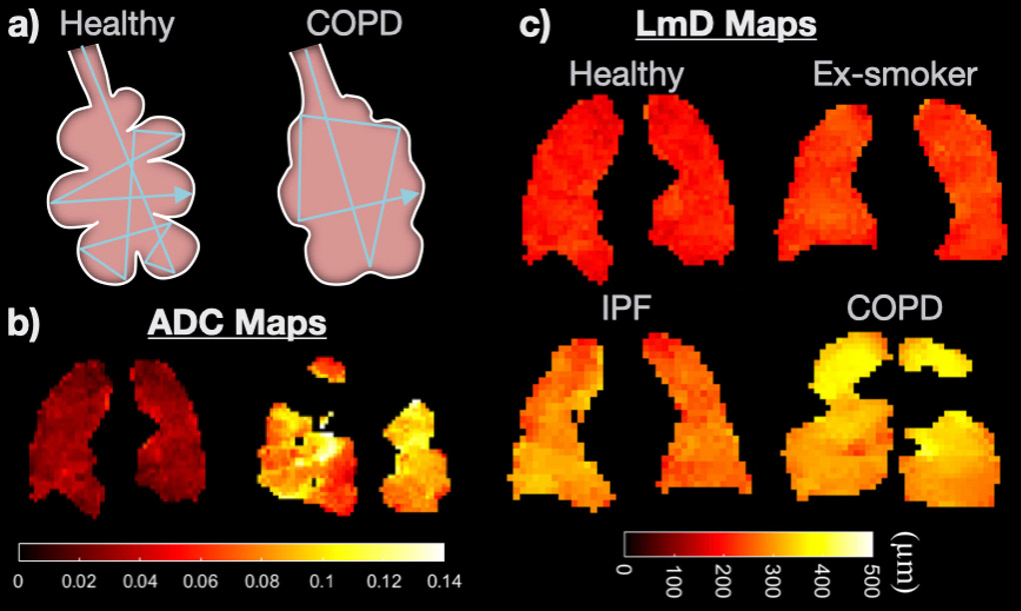
(a) cartoon of HP gas diffusion in the lungs of a healthy subject and a patient with COPD; diffusion is more *restricted* in the former, while emphysematous tissue destruction leads to *less-restricted* (*freer*) diffusion in the latter, *i.e*. increased ADC. (**b**) HP ^129^Xe ADC maps, indicating significantly increased ADC in a patient with COPD compared with a healthy subject. (**c**) representative diffusion-weighted HP ^129^Xe MRI-derived morphological maps of the mean alveolar diffusion length scale, depicting increased alveolar size in patients with IPF and COPD in comparison to healthy subjects (adapted from^8^). As is clear from the ADC and LmD maps obtained from the lungs of the COPD patient in b) and c); these metrics can only be calculated for ventilated areas of the lung. ADC, apparent diffusion coefficient; COPD, chronic obstructive pulmonary disease; HP, hyperpolarised; IPF, idiopathic pulmonary fibrosis.

Microstructure (Diffusion-weighted MRI): Both ^3^He and ^129^Xe gases are 4—5 orders of magnitude more diffusive than water molecules in bodily tissues, and are therefore well suited for diffusion-weighted MRI. Inhaled gas atoms diffuse in the acinar airspace through random Brownian motion, and encounter the alveolar walls several times over a time-scale of ~milliseconds (Figure 2a). This leads to a diffusion restriction dependent on the acinar microstructure, and which can be probed by HP gas diffusion-weighted MRI.

Biomarkers of microstructure: The apparent diffusion coefficient (ADC) can be mapped voxel-by-voxel providing regional information on alveolar airspace size^9^; its mean value across the lungs provides a simple, sensitive biomarker (Figure 2b). Multiple b-value diffusion-weighted MRI with theoretical modelling of the complex gas diffusion signal allows quantification of various acinar airway morphological parameters including the surface-area-to-volume ratio, alveolar radii and mean diffusive length scale (LmD) (Figure 2c).^10–12^

**Figure 3.**
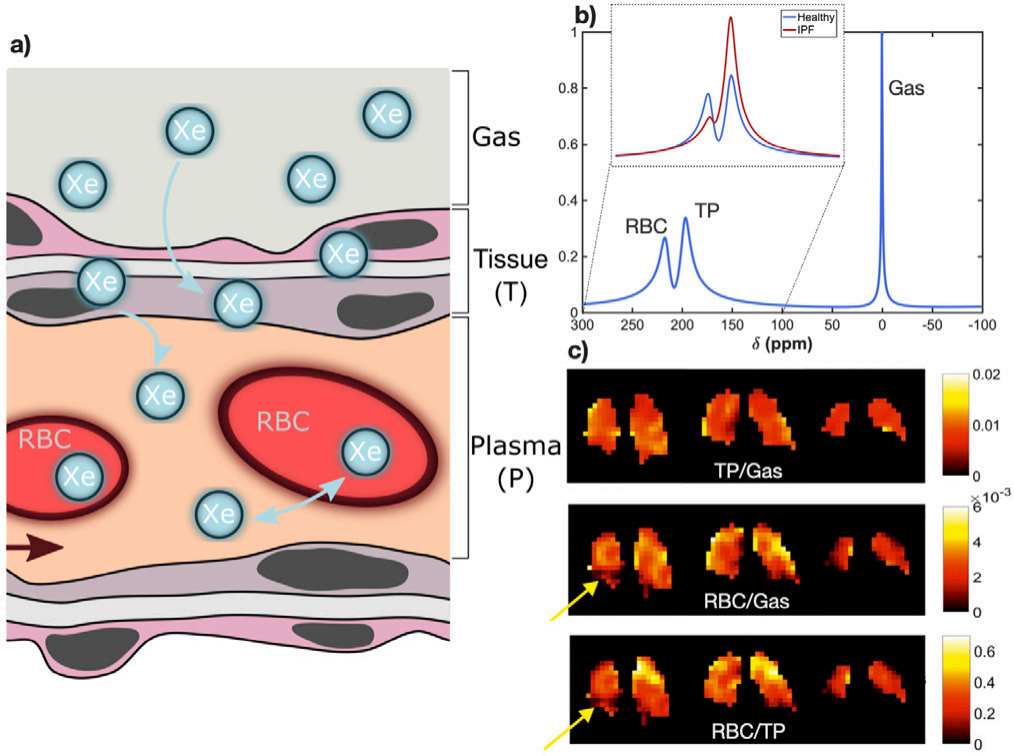
a) Schematic of xenon diffusion from the alveolar (gas) phase into the lung tissue and blood. (**b**) Simulated ^129^Xe MR spectra in healthy human lungs, indicating the chemically-shifted (δ) gas, TP and RBC resonances. In the inset, the difference in the dissolved phase (TP and RBC) part of the spectrum typically observed in the lungs of patients with IPF compared with healthy subjects is shown; notably, an increased TP resonance relative amplitude and reduced RBC resonance relative amplitude. These chemically shifted signals can be separately imaged via chemical shift imaging pulse sequences to obtain signal ratio maps; **c**) representative signal ratio maps obtained from a patient with IPF; yellow arrows indicate a region of reduced RBC transfer (gas exchange) that was normally ventilated (reproduced from Collier et al.^13^). IPF, diopathic pulmonary fibrosis; RBC, red blood cell; TP, tissue and blood plasma.

Gas exchange (Dissolved-phase ^129^Xe MRI): Xenon is moderately soluble in lung parenchyma and blood (Figure 3a) and ^129^Xe exhibits distinct resonances in the tissue and blood plasma (TP) and separately, the red blood cells (RBCs) in the pulmonary capillaries that are chemically shifted from the gaseous ^129^Xe in the alveolar airspace.^14^ MR spectroscopy (MRS),^15,16^ chemical shift saturation recovery (CSSR)^17^ and chemical shift imaging techniques^13,18^ allow the investigation of gas exchange function by directly measuring the ^129^Xe MR signal in each of these compartments.

Gas exchange biomarkers: The signal ratios of ^129^Xe in different physiochemical compartments; RBC:TP, RBC:Gas, TP:Gas are used as quantitative gas exchange biomarkers (Figure 3b), and can be used to distinguish RBC transfer (indicative of *true* gas exchange) from parenchymal tissue thickening. Diffusion modelling of time resolved ^129^Xe spectroscopic techniques allows quantification of various alveolar morphological parameters.

A summary of the different imaging biomarkers that can be derived from HP gas MRI, and their reference values in healthy subjects, is presented in Table 1.

**Table 1.** Summary of key clinical metrics that can be derived from HP gas MRI

Biomarker	Description	Healthy reference balues
VDP (VV)	Ventilation defect percentage (Ventilated volume): percentage of total lung volume that is not ventilated (or opposite) → metric of ventilation	0 ~ 5%^b^ (95 ~ 100%) (ref^5,19^)
CV (VH_I_)	Coefficient of variation (Ventilation heterogeneity index): metrics of regional ventilation heterogeneity	Mean CV <15% (ref^20^) IQR CV <10% (ref^5^) ^*c*^
ADC	Apparent diffusion coefficient: describes how far gas can diffuse in a given time before being impeded → metric of alveolar size	^3^He: 0.190 ± 0.017 cm^2^.s^−1^ ^129^Xe: 0.038 ± 0.003 cm^2^.s^−1^ (ref^6^)
LmD	Mean diffusive length scale: comparable to histology mean linear intercept → metric of alveolar size	^3^He: 212 ± 24 µm ^129^Xe: 205 ± 23 µm (ref^8^)
RBC/TP RBC/Gas TP/Gas	Ratios of ^129^Xe signal in RBC *vs* tissue plasma *vs* gas phase (alveoli) of the lungs: RBC/TP: metric of gas exchange function and parenchymal tissue thickening; RBC/Gas: metric of gas exchange and perfusion; TP/Gas: metric of tissue thickening	~0.47 ~3.6×10^−3^ ~7.5×10^−3^ (ref^13^ ^a^)

ADC, apparent diffusion coefficient; CV, coefficient of variation; IQR, interquartile range; ṢNR, signal-to-noise ratio; RBC, red blood cell; TP, tissue and blood plasma; VDP, ventilation defect percentage; VH_I_, Ventilation heterogeneity index; VV, ventilated volume.

a These values are appropriate for the IDEAL method described in Collier et al^13^ (for other gas exchange imaging methods, values may differ, see *e.g*. Wang et al^21^)

b These values depend on the exact analysis technique and are provided as a guide only.

c These values vary with analysis technique, image SNR, and statistic (mean, median, IQR, etc.) chosen

### Practicalities of HP gas MRI

From a practical perspective, the following requirements must be met to perform HP gas MRI in a clinical setting: (1) regulatory-approved gas polariser (>£350k); (2) licence for gas manufacture and inhalation (may initially be a research licence as an investigative medicinal product, but ultimately, approval from the MHRA, FDA or other regulatory body is needed for diagnostic use); (3) multinuclear (broadband) MRI scanner (1.5 T or 3 T) and vendor support; (4) radiofrequency transmit-receive coils for the nuclei of interest (>£35k); (5) NHS or other healthcare institution referral pathway.

From the perspective of raw materials, ^129^Xe-isotope enriched xenon costs around £180/L and doses of 500—1000 mL are typically needed. However, use of the 26% ^129^Xe natural abundance mixture is much cheaper (~£25/L) and provides adequate results for ventilation imaging (the most common clinically-requested scan in our institution).^22^ Whilst the initial outlay for several of these components can be significant, a scalable health-care economics model for patient scanning with HP ^129^Xe MRI is potentially achievable in the context of a regional hub in a large teaching hospital setting, when viewed alongside the NHS tariff costs for other specialist imaging and comprehensive lung function testing.

A HP gas lung MRI protocol can take around 15—30 min to complete, including the time required for set-up of the ^3^He or ^129^Xe radiofrequency coil. In total, between 2 and 5 doses of gas are typically delivered; one small dose (~few 10 of mL) for calibration of the acquisition parameters – including flip angle and Larmor resonance frequency of the gas nuclei – and the remaining main dose(s) (500—1000 mL) for ventilation, diffusion-weighted and/or dissolved-phase imaging. The choice of scans to perform depends on the diagnosis/symptoms of the patient, as discussed in the following sections. For example, in asthma, which is characterised by airway inflammation, ventilation imaging alone is usually sufficiently sensitive to airway obstruction and additional HP gas acquisitions may not add clinically significant information. In contrast, acquisition of dissolved-phase imaging data will be of a priority in patients where gas-exchange limitation is known/anticipated. The HP gas protocol is usually combined with a ^1^H pulmonary MRI protocol, which can take an additional 10—30 min to complete and may include some or all of the following: anatomical scans including spoiled gradient echo-, turbo spin echo or steady-state free precession-based sequences; ultrashort echo time scans for high-resolution structural imaging; non-contrast and/or contrast-enhanced perfusion scans; dynamic ^1^H MRI and possibly oxygen-enhanced imaging. In our experience, these methods can be performed equally well on both 1.5 T and 3 T scanner platforms that are typical for the bulk of clinical practice, with the former generally more forgiving for thoracic applications.^22–25^ As the HP gas portion of the scan is most time critical (due to the dose timing of the hyperpolarisation process) and costly to repeat, it is conventional to perform this prior to the ^1^H portion and certainly before any paramagnetic intravenous contrast agents have been administered. The signal enhancement induced by the hyperpolarisation process is non-permanent and decays according to the longitudinal relaxation time (T_1_) of the gas. This is of the order of hours in an oxygen-free environment and a stable magnetic field, but reduces to ~10s of seconds in the lungs. Ideally, doses of HP gas should be stored in a magnetic field prior to delivery; it is practical to situate the polariser in a room proximal to the MR scanner or otherwise use a magnetic container to transport the gas over larger distances.

Both ^3^He and ^129^Xe are considered safe for inhalation in the relatively small dosages used for MRI. In 100 individuals with a range of lung conditions, Lutey et al reported no serious adverse events and no effect on vital signs from the ^3^He breath-hold MRI procedure, other than a small post-imaging decrease in mean heart rate and a transient mean decrease in SpO_2_ of ~4% within the first minute after inhalation.^26^ Unlike helium, xenon has anaesthetic properties at a sustained minimum alveolar concentration (MAC) of between 63 and 71%,^27,28^ however the doses and short breath-hold durations used in HP ^129^Xe lung MRI yield a transient alveolar concentration far below this. A number of safety and tolerability studies in adults and children with a variety of pulmonary disorders have reported no serious or severe adverse events after ^129^Xe breath-hold.^29–31^ As with ^3^He, transient decreases in SpO_2_ of a few percentage points after ^129^Xe breath-hold are commonly observed, but resolve within 1—2 min. Many adult patients report mild transient symptoms including dizziness, paresthesia and euphoria that fully resolve within a few minutes after inhalation.^29^

We note that a significant proportion of the historical literature is occupied by research on ^3^He due to its relatively high gyromagnetic ratio (high intrinsic MR signal). However, ^3^He is not naturally abundant, its availability has become severely regulated, and costs have risen to the point that it is not an economically viable agent for widespread clinical use.^32^ For this reason, coupled with the fact that dissolved-phase MRI of pulmonary gas exchange is exclusively possible with ^129^Xe, which is of great clinical interest, the HP gas MRI field has generally transitioned to the use of ^129^Xe over the last 5—10 years. A comparison of the key properties of the two gases relevant to their application in lung MRI is shown in Table 2; most notably highlighting the lower diffusivity of ^129^Xe.

**Table 2.** Key Properties of HP Gas Nuclei

Property	^1^H	^3^He	^129^Xe
Isotopic abundance (%)	99.99	1.4 × 10^−4^	26.44 (natural abundance) 80—90 (^129^Xe-enriched)
Gyromagnetic ratio (MHz/T)	42.58	−32.44	−11.78
Self-diffusion coefficient (cm^2^/s)^*a*^	2 × 10^−5^	2.05	0.06
Diffusion coefficient in air (cm^2^/s)^*a*^	–	0.86	0.14
Approximate Cost (£/L)	–	500	150 (^129^Xe-enriched) 25 (natural abundance)

a Diffusion coefficients taken from Chen et al^33^

In the following sections, we review the respiratory disease areas where the technique has made a clinical research impact.

### COPD & emphysema

Chronic obstructive lung disease (COPD) is a leading cause of mortality worldwide. Underdiagnosis, comorbidities and a lack of treatment access, all contribute to a significant healthcare burden.^34^ Emphysema is a form of COPD characterised by irreversible damage to the alveolar walls, leading to impaired gas exchange. Hyperpolarised gas MRI offers a sensitive means to characterise and stage emphysema, and may provide clinical utility in both early and late disease; in identifying early disease and suitable patients for early interventions, and also guiding targeted therapies such as endobronchial valves and lung volume reduction surgery.

### Ventilation

HP gas ventilation MRI is highly sensitive to airway obstruction, and exhibits significant ventilation abnormalities in patients with emphysema, and COPD more broadly. The safety of HP gas ventilation MRI in smokers, patients with COPD and candidates for lung volume reduction surgery (LVRS) is well documented.^26,29^ ^129^Xe tends to exhibit increased VDP in participants with COPD when compared with^3^He,^6,35^ and regions of high ^129^Xe VDP have been reported to correlate with emphysema on CT.^36^ Direct visualisation of collateral ventilation – a proposed response mechanism to compensate for airflow obstruction – has been reported using HP ^3^He.^37^ In a three-centre study, HP ^3^He ventilation MRI correctly categorised patients with COPD and revealed structure-function abnormalities upon comparison to CT.^38^ Complementary use of HP gas ventilation MRI and CT identified basal-lung predominant ventilation defects and apical-lung predominant CT emphysema, with utility for characterisation of COPD grades.^39^ VDP is more sensitive to bronchodilator therapy in COPD than the gold-standard spirometry measurement of airflow obstruction; FEV_1_ (forced expiratory volume in 1 sec).^40^ Furthermore, HP gas ventilation MRI can depict the regional heterogeneity in bronchodilator response over the lungs, whereas spirometry measurements only provide information on the whole-lung *average* function. Moreover, HP gas MRI ventilation metrics show improved sensitivity to longitudinal lung function decline in COPD when compared to FEV_1_.^41^ In a comprehensive single-site study of several MRI and CT biomarkers, only VDP longitudinal change correlated with St. George’s Respiratory Questionnaire on COPD quality of life.^42^ HP gas ventilation MRI is predictive of exacerbations in mild/moderate COPD^43^ and when combined with other imaging metrics such as ADC (see below), is predictive of FEV_1_ decline in smokers.^44^ Quantitative ventilation MRI with HP gases holds some promise for guiding LVRS^45^ and via the visualisation of collateral ventilation, is likely to have clinical utility for this purpose in the future. Moreover, the method may be useful in aiding the differentiation of asthma from COPD patients with pre- and post-bronchodilator reversibility ventilation imaging^46^ alongside spirometric evaluation.

### Alveolar microstructure

In patients with emphysema, alveolar tissue destruction leads to less-restricted (freer) diffusion and in-turn, an elevation in the global mean value of ADC; both ^3^He^9,47^ and ^129^Xe^6,35,48^ ADC values are approximately twice that in healthy lungs. HP gas diffusion biomarkers agree well with the histologically-derived alveolar mean linear intercept (Lm) – the gold-standard measurement of alveolar size – in *ex vivo* human lungs.^49–51^ In addition, significant correlations between diffusion biomarkers and existing clinical measures for diagnosing and quantifying emphysema,^52–55^ including FEV_1_,^6,47,56^ transfer factor of the lungs for carbon monoxide (TL_CO_)^6,35,48^ and quantitative CT measures^35,56–58^ have been reported. When compared to CT mean lung density and emphysema index (−950 HU), ADC is more effective in separating patients with COPD and healthy controls, and correlates more strongly with TL_CO_.^38,59^ The sensitivity of HP gas diffusion-weighted MRI in detecting early/mild emphysematous lung disease has clinical promise. Diffusion biomarkers are significantly elevated in ex-smokers with COPD compared to age-matched never-smokers.^56,60–62^ Moreover, asymptomatic smokers (with normal spirometry) demonstrate subclinical differences to never-smokers; more heterogeneous distribution of ADC^62,63^ and reduction in alveolar sleeve depth (outer radius of the alveolar shell when modelling acinar airways as cylinders).^61^ Diffusion biomarkers also demonstrate sensitivity to age-related acinar changes or senile “emphysema”^64^ and show increased alveolar enlargement from childhood^65^ to adulthood.^66–68^ HP gas diffusion-weighted MRI is well-suited to longitudinal monitoring of COPD/emphysema disease progression as it is non-ionising. In ex-smokers with COPD, significant increases in ADC were observed after 2 years in the absence of significant change in FEV_1_.^41^ The development of quantitative metrics such as a HP gas MRI emphysema index^58^ shows promise for longitudinal studies as an easily-interpretable metric of emphysema severity with comparable diagnostic performance to CT-based emphysema indices and TL_CO_. Recent developments in spatial co-registration of images from HP diffusion-weighted MRI and CT have facilitated quantitative multi parametric response mapping (mPRM),^69^ which has revealed subclinical emphysema and small airways disease in ex-smokers without COPD that was not detectable with CT or MRI alone.^70^

### Interstitial lung disease

Interstitial lung disease (ILD) includes a heterogenous range of chronic lung conditions characterised by inflammation and/or scarring of the lung interstitium. Idiopathic pulmonary fibrosis (IPF) – one of the most common ILDs – is a progressive, ultimately fatal disease of unknown aetiology. ILD is usually characterised as a restrictive lung function disorder most commonly assessed by the spirometry metric, forced vital capacity (FVC). Imaging – particularly high-resolution CT (HRCT) – plays a key role in the diagnosis of IPF and its distinction from other ILDs. However, the reproducibility and sensitivity of FVC and TL_CO_ to detect lung disease remains challenging, and most CT scans remain qualitative. HP ^129^Xe MRI, alongside quantitative HRCT is poised to play an important future role offering objective, reproducible, and sensitive imaging biomarkers, in the monitoring of ILD progression, prognosis and assessment of response to novel treatments.

### Gas exchange

HP ^129^Xe MRS provides sensitive global metrics of gas exchange, such as the ratio of ^129^Xe MR signal in the RBCs to that in the TP (RBC:TP), which is significantly reduced in patients with IPF compared to healthy volunteers (*p* < 0.0002)^16^ and strongly correlates with TL_CO_. A pilot study using a ^129^Xe time-resolved spectroscopic technique reported a statistically significant difference in alveolar septal thickness between healthy volunteers and patients with IPF and scleroderma (SSc), but no distinction between patient groups.^17^ Recently, ^129^Xe MRS was reported to have improved sensitivity to 12 month change in patients with IPF (*p* = 0.001) compared to FVC (*p* = 0.048) and TL_CO_ (*p* = 0.881).^71^ Whilst MRS provides a simple, sensitive global metric, regional gas exchange impairment in IPF can be visualised by HP ^129^Xe spectroscopic imaging methods.^13,21,72^ Regions of reduced RBC transfer (RBC:Gas or RBC:TP) are observed predominantly in peripheral and basal lung regions, corresponding spatially with fibrosis on CT^18,72^ though correlation with CT fibrosis scoring has been weak to date.^72^ HP ^129^Xe gas exchange imaging metrics correlate strongly with TL_CO_^13,21,72^ and show sensitivity to longitudinal IPF disease progression.^73^ HP ^129^Xe MRI offers a means to discriminate gas exchange impairment resulting from tissue thickening (TP:Gas) and other mechanisms, and has been utilised to characterise cardiopulmonary function in a range of disorders including COPD, IPF, left heart failure (LHF) and pulmonary arterial hypertension (PAH).^74^ A novel means to quantify cardio-pulmonary-vascular involvement via detecting cardiogenic oscillations in ^129^Xe RBC MR signal by spectroscopy^15^ and imaging^75^ has revealed increased modulation of RBC signal in IPF.^13,75^ Increased RBC signal oscillations were also found in patients with LHF, suggesting changes in capillary blood volume during the cardiac cycle and secondary to post-capillary PH.^74^

There have been no published reports of the use of HP gas ventilation MRI in ILD, however, we note that gas exchange imaging techniques inherently acquire (low-resolution) ventilation images without the need for a separate breath-hold. Recently, HP ^3^He gas diffusion-weighted MRI revealed that both ADC and LmD correlate with TL_CO_, carbon monoxide transfer coefficient (K_CO_) and regional fibrosis on CT in patients with IPF.^76^ LmD increased significantly over 12 months, whilst other metrics did not. Increased ADC and LmD measurements may reflect reduced acinar integrity due to microstructural changes in the lung, secondary to fibrosis. We anticipate the complementary use of HP gas MRI techniques alongside novel methods for quantitative CT that show prognostic value in IPF,^77^ to reveal the mechanisms behind the observed changes in alveolar microstructure, and further understand gas exchange structure-function characteristics. Furthermore, the unique ability to measure gas transfer limitation when combined with DCE perfusion MRI for direct quantitative assessment of lung perfusion, provides unique insight in to distinguishing diffusion block from perfusion deficit. This powerful combination will be of use in phenotyping the overlap of interstitial and pulmonary vascular lung pathophysiology, and recent pilot studies have shown these methods to be sensitive to such mechanisms in post-COVID lung disease.^78^

### Asthma

One of the most common respiratory conditions worldwide, asthma is a chronic airway disease that accounts for a UK health-care burden of at least £1.1 billion each year.^79^ Clinical efforts are focused on improving patient management and preventative medicine.^80^ There is significant variability in the clinical, physiological and pathological presentation^81^ and different phenotypes can have different responses to therapy.^82^ In asthma, hyperpolarised gas MRI offers a unique, sensitive means to visualise the extent of airways disease, its reversibility, and the functional response of the lungs to therapy and may find future utility in assessment of novel biologics for personalised medicine.

Ventilation defects visualised by hyperpolarised gas MRI, depicting regions of airflow obstruction in asthma, increase with clinical asthma severity,^83,84^ and are associated with age, airway hyperresponsiveness and airway remodelling.^85^ Several studies have reported correlations between numbers of ventilation defects (or VDP) and spirometric indices, including FEV_1_^83,84,86–89^ and FEV_1_/FVC.^83,84,86,87,89,90^ VDP increases after methacholine (bronchoprovocation) challenge^91^ or exercise^90^ and decreases after bronchodilator inhalation.^91^ Ventilation defects have also been associated with increased airway resistance,^85,91^ fractional exhaled nitric oxide (FeNO; a marker of inflammation)^85,91^ and lung clearance index (LCI), a measure of ventilation heterogeneity.^92^ Patients with severe, poorly-controlled asthma and low quality of life have been reported to have increased ventilation heterogeneity and VDP,^92^ and in a larger population with a wide range of severity, VDP correlated inversely with asthma control.^87^ Increased VDP is associated with asthma exacerbations, leading to hospitalization (including in patients with mild/moderate disease)^86^ and with exacerbation frequency over 2 years following MRI.^87^

HP gas MRI ventilation abnormalities have also been reported to correlate with blood eosinophil count,^86,87^ post-bronchodilator sputum eosinophils,^93^ and more invasive, yet sensitive metrics such as localised bronchoscopy and neutrophils in bronchoalveolar lavage fluid.^94^ HP gas MRI and CT provide highly complementary information in the identification of structure-function phenotypes of asthma. In particular, regions of air trapping^94^ and mucous plugging^95^ on CT have been reported to show significant overlap with ventilation defects. In a recent study, higher VDP in patients with >10 missing airway subsegments quantified by CT total airway count was reported.^96^ Several studies have revealed a spatial heterogeneity in location of ventilation abnormalities in patients with asthma at baseline,^84,90,94^ and increased abnormalities in posterior regions following exercise.^90^ Despite the variable nature of airflow obstruction in asthma, the location of ventilation defects often persists over time.^97^ A recent 6 year follow-up study found that ventilation defects remained localised, and did not significantly change in size in ~70% of patients with asthma; in the remaining ~30% of patients, defects were larger at follow-up.^98^

HP gas MRI has been utilised to visualise not only response to bronchodilator,^89,99^ but also to novel and existing treatments, including bronchial thermoplasty^100^ and the anti-inflammatory drug montelukast.^90^ A recent report demonstrated that HP gas ventilation MRI can be used to guide bronchial thermoplasty treatment.^101^ In the past year, the first report of HP gas MRI to assess biologic treatment of persistent post-bronchodilator ventilation abnormalities^93^ in severe asthma with uncontrolled sputum eosinophilia was published^102^; a first step towards HP gas MRI for personalised medicine. In our recent experience of using ^129^Xe ventilation MRI clinically in a real-world population of patients with difficult asthma, we found that it provided unique information on disease severity and bronchodilator reversibility, which aided in the clinical evaluation of asthma.^103^ Evidence of airways obstruction on MRI supported the use of further treatment in patients where the clinical picture was unclear, whilst conversely, well-preserved ventilation on MRI alongside poor spirometry and/or symptom control suggested the possibility of coexisting breathing control issues or laryngeal disorders.

Paediatric asthma is likely to be a future area of research focus.^104^ Of particular note, the safety and tolerability of HP gas MRI in 66 children with asthma reported no serious adverse events and three minor adverse events (2.3%; including headache, dizziness and mild hypoxia).^105^ Preliminary reports have shown that VDP and the number of defects per slice are predictive of asthma outcomes, including clinical asthma severity, corticosteroid use, and health-care utilisation.^106,107^ We anticipate future use of HP gas ventilation MRI in the identification of patients prone to exacerbations, and/or those suitable for personalised treatments.

### Cystic fibrosis

Cystic fibrosis (CF) is a hereditary disease caused by a mutation in the cystic fibrosis transmembrane conductance regulator (CFTR) gene. Lung disease is the primary cause of morbidity and mortality in patients with CF, and is progressive throughout life, beginning soon after birth. Significant scientific breakthroughs have enabled the production of highly effective CFTR modulator therapies that are now becoming available for a large proportion of patients,^108^ however, the health-care burden of CF remains high, and clinical care is often complex. There is a clinical need to be able to identify early changes in lung disease to prevent further deterioration. Hyperpolarised gas MRI techniques may be ideally placed to identify early disease and moreover provide a sensitive, safe means to monitor longitudinal disease progression and therapy response.

HP gas ventilation MRI procedures are safe and well-tolerated in adult and paediatric patients with CF.^31^ Ventilation abnormalities in patients with CF have been investigated by both static^109^ and dynamic^110^ MRI and abnormalities typically present as heterogeneous and patchy. The technique is repeatable, with a minor change in VDP on the same day^111^ and at 7 day and 4 week follow-up, and ventilation defects located in the same spatial regions.^112,113^ Zonal analysis of ventilation MRI metrics shows good agreement with HRCT score in adults with CF.^114^ CF exhibits several characteristic structural abnormalities (*e.g.* mucus plugging, bronchiectasis, bronchial wall thickening) and recently, some agreement in the spatial location of structural ^1^H MRI and functional ^129^Xe ventilation MRI abnormalities has been reported.^115^ When combined, these methods may offer a non-invasive means to predict clinical outcomes in paediatric CF.^116^ VDP exhibits a strong correlation with LCI,^5,117^ a metric of ventilation heterogeneity – derived from the multiple breath washout (MBW) pulmonary function test – that shows greater sensitivity than conventional spirometry in the detection of mild CF.^118^ Furthermore, an analogous metric of ventilation heterogeneity to LCI (a so-called ventilation heterogeneity index (VH_I_)) can be derived from HP gas ventilation MRI, and exhibits good agreement with MBW metrics including LCI.^5^ Recently, an imaging analogue of the MBW pulmonary function test has been shown to be feasible using multiple breath HP gas MRI in CF.^119^

Ventilation abnormalities on HP gas MRI appear to be a characteristic of the earliest measurable changes to lung function in CF. Several studies have demonstrated how in patients with clinically stable lung disease and normal values for FEV_1_, that ventilation abnormalities are already present.^20,117,120^ The sensitivity of ventilation MRI to CF pathophysiology has been found to be superior to CT, conventional MRI or LCI, and in several cases, ventilation abnormalities on MRI in the absence of abnormality on CT have been observed.^20^ Several studies have reported the high sensitivity of HP gas ventilation MRI to detect a therapeutic response in CF, such as bronchodilator and airway clearance treatment,^121^ chest physiotherapy,^122,123^ exercise,^124^ antibiotics^125^ and ivacaftor,^126^ and we anticipate clinical utilisation in this manner in the future. Moreover, recent reports have highlighted the high sensitivity of HP gas ventilation MRI to mild functional change in CF over a 1—2 year period where spirometry (FEV_1_) showed no significant change.^111,127^ In particular, VDP was found to have a higher median longitudinal change than LCI and FEV_1_ and relative changes in VH_I_ significantly correlated with those of LCI.^111^ In the same report, based on observational follow-up of CF patients at an average of 16 months, thresholds for significant clinical changes in VDP were reported, as well as estimates for clinical trial population sizes.^111^

### Clinical utility: our preliminary experience

HP gas MRI holds the potential to play a clinical role in a range of pulmonary conditions including and additional to those listed above. In 2015, our centre was authorised by the UK MHRA to manufacture HP gases for clinical MRI indications, and since then we have had more than 500 referrals from clinicians around the UK, to assist with difficult diagnoses and provide additional clinical information. Qualitative imaging data is generated as a technical report (see Supporting Information) that is used as the basis of a multidisciplinary team (MDT) meeting composed of: respiratory physicians, adult/paediatric chest radiologists, MRI physicists, physiologists and radiographers, and from which a qualitative radiological report is also drawn. In the future, defining an accurate clinical reporting terminology of these images will likely require consensus-building work with systematic qualitative radiological interpretation of the imaging features observed. In terms of clinical reporting, we believe it is critical to report HP gas MR imaging biomarkers as non-subjective, quantitative metrics, and, for clinical utility, to ensure that images are interpreted within an MDT setting so that all staff involved build-up experience with the images and metrics.

In the following, we present several patient case studies that highlight the clinical utility of the HP gas method.

### Paediatric lung disorders

**Figure 4.**
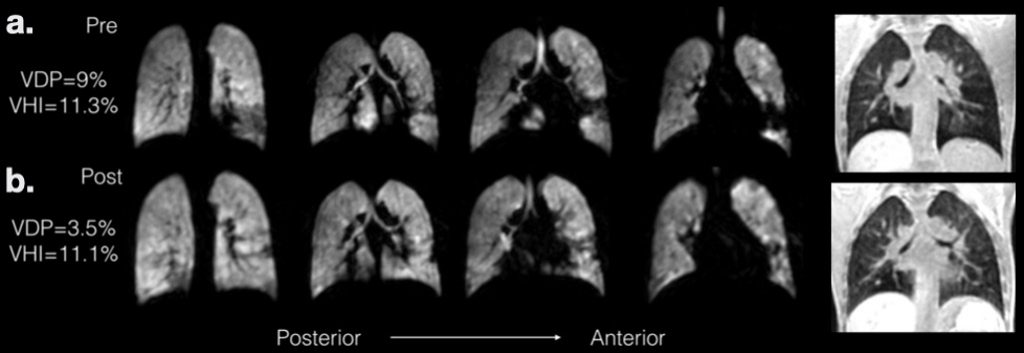
Patient A (13 years): clinical diagnosis of PCD; referred due to questionable clinical benefit of their standard physiotherapy. At baseline, spirometry showed a mixed obstructive/restrictive defect, suggesting an obstructive pattern with a significant gas-trapping component. Pre-physiotherapy ^129^Xe ventilation images (**a**) show clear evidence of ventilation defects in the lung bases at baseline. Physiotherapy was performed using a PEP device. Post-physiotherapy ^129^Xe MRI (**b**) exhibited a marked reduction in the degree of ventilation defects, although some abnormalities remained. Whilst VDP decreased from 9.0 to 3.5%, there was no clear change in VH_I_. Post-physiotherapy spirometry showed an increase in FEV_1_ of 8.4%, but no change in FVC. Complementary ^1^H SPGR imaging at RV (far right panel) indicated a visible decrease in gas trapping in the locations where ventilation defects improved, though some remaining gas trapping was evident. Complementary structure-function (^1^H-^129^Xe) MRI helped assess the benefits of performing the physiotherapy regime in this patient. Despite small improvements in FEV_1_, a clear improvement in regional ventilation and a significant reduction in VDP was observed. FVC, forced vital capacity; PEP, positive expiratory pressure; VDP, ventilation defect percentage.

**Figure 5.**
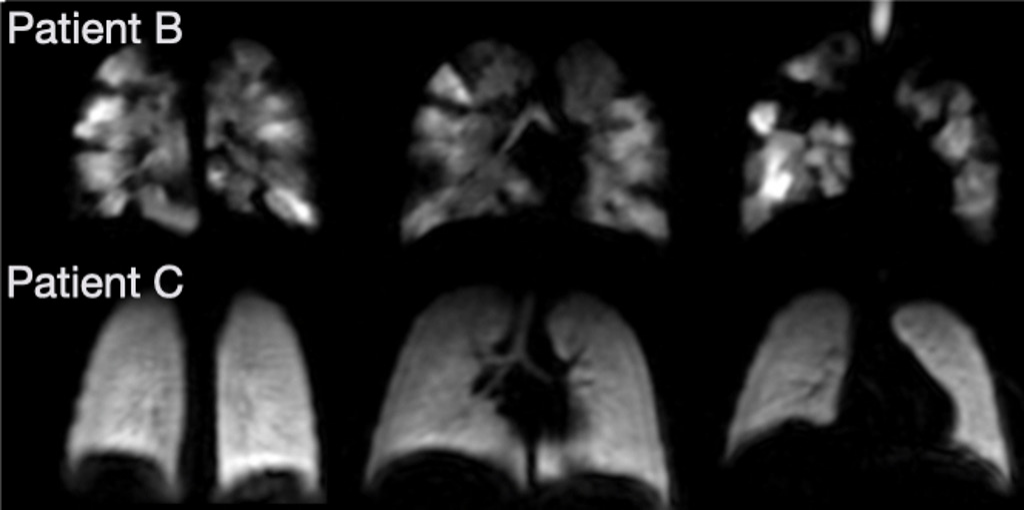
Patients B and C: Two patients with a clinical diagnosis of Fanconi Anaemia who had previously received a bone-marrow transplant. Both patients presented with similar symptoms of shortness of breath on exertion. Due to the underlying diagnosis and ionising radiation risk, the clinical team were reluctant to request a clinical CT. Both patients reported respiratory symptoms. Patient B (5 years) was unable to perform spirometry with adequate technique. Patient C had normal spirometry and TL_CO_. These patients exhibited quite different patterns of underlying ventilation; Patient B had significant ventilation defects present throughout the lungs, whilst Patient C exhibited homogeneous ventilation. Patient B therefore had underlying respiratory pathology matching the documented symptoms, whilst Patient C had normal spirometry, T_LCO_ and ^129^Xe ventilation MRI. The MR images in these two similar cases demonstrated different outcomes and allowed bespoke management plans to be created.

**Figure 6.**
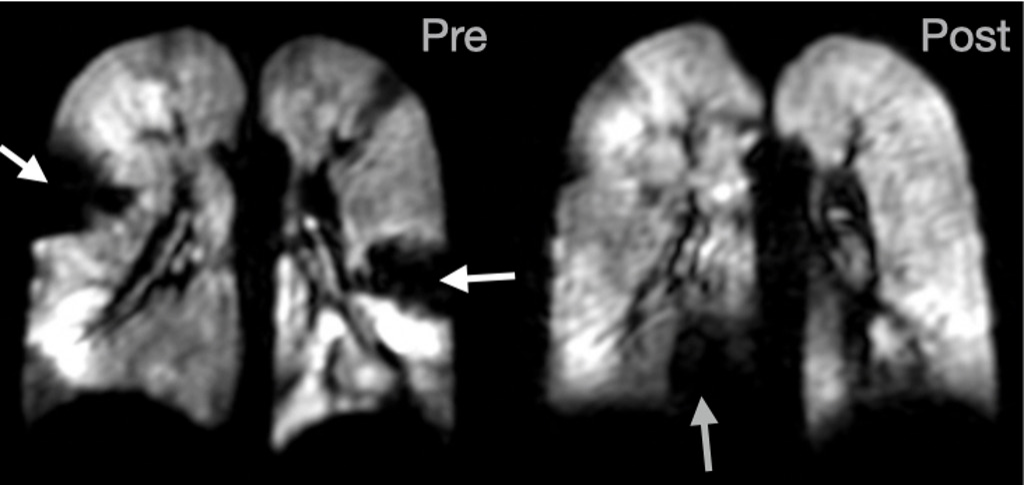
Patient D: clinical diagnosis of non-CF bronchiectasis. The patient suffered from a chronic productive cough and amongst other treatments had a two-week course of IV antibiotics three months prior to referral for MRI. The patient’s FEV_1_ was static at >90%-predicted and historically did not change after antibiotics, making it difficult to determine the treatment efficacy. The patient was therefore referred for pre- and post-therapy ventilation MRI during their next two-week course of IV antibiotics. Large ventilation defects were evident in both lungs in baseline ventilation images (FEV_1_ = 94%). Post-therapy, the patient’s FEV_1_ decreased to 89%-predicted, whilst ventilation images exhibited almost complete resolution of the large ventilation defects that were present at baseline (white arrows), although a new ventilation defect was present in the basal right lung (grey arrow). These images helped reassure the clinical team as to the efficacy of the current treatment regime. This case highlights the sensitivity and benefit of regional lung function assessment offered by HP gas MRI in children on therapy, and the relative insensitivity of FEV_1_ to detect these changes. CF, cystic fibrosis;

**Figure 7.**
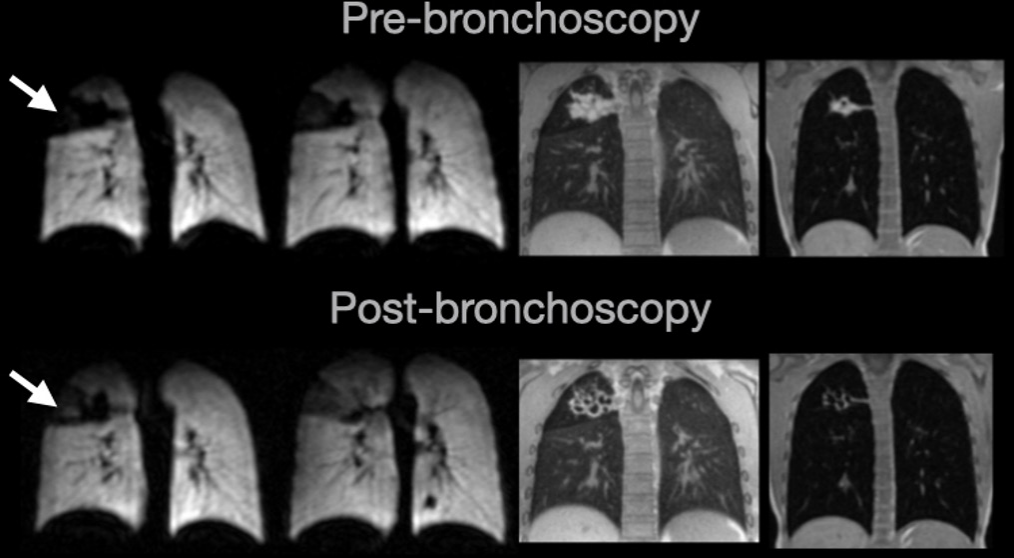
Patient E: clinical diagnosis of CF monitored longitudinally. At the patient’s routine clinical review, a chest X-ray and CT showed a right-upper lobe collapse and the patient was diagnosed with ABPA. After standard clinical management, a repeat CT scan at 6 months was performed, which still showed the right-upper lobe collapse. At this stage, the patient had never reported any clinical symptoms and spirometry had always remained unchanged at 90—95% predicted. The patient was therefore referred for MRI assessment of both ventilation and structure. A large ventilation defect was present in the right-upper lobe on ventilation MRI and did not ventilate at TLC, suggesting complete obstruction. ^1^H MRI images depicted the cause of the ventilation defect; a large mass of mucus-filled airways in the right-upper lobe. Upon reviewing these images, the clinical team decided to perform a bronchoscopy and repeat the MRI assessment. Repeat MRI was performed approximately 2 weeks post-bronchoscopy and showed that the large ventilation defect remained at end inspiratory tidal volume, but with some improved ventilation compared with pre-bronchoscopy, especially at TLC. ^1^H structural MRI demonstrated the removal of a large mass of mucus, though the mucus-affected airways remained damaged and functionally impaired. This case study highlights the complementary nature of ^129^Xe functional and ^1^H structural MRI and their promise in clinical assessment of bronchoscopy success as an alternative to repeat CT, especially when spirometry has limited clinical value. Key: from left-to-right; ^129^Xe ventilation MRI at end inspiratory tidal volume, ^129^Xe MRI at TLC, ^1^H ultra-short echo MRI, ^1^H spoiled gradient echo MRI at TLC. ABPA, allergic bronchopulmonary aspergillosis; CF, cystic fibrosis; TLC, total lung capacity.

Of our referrals to date, around 20% have been paediatric patients. Many of these patients had limited clinical imaging prior to referral due to concerns over ionising radiation exposure. Example clinical case studies are shown in the following figures (Figure 4, Figure 5, Figure 6, Figure 7).

### Adult lung disorders

**Figure 8.**
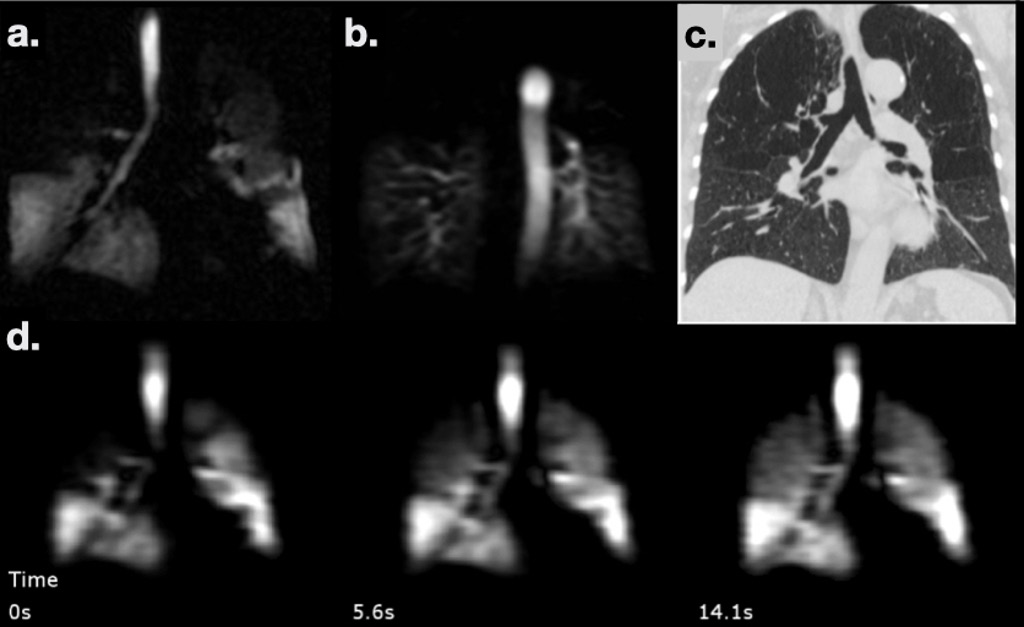
Patient F: LVRS case study. 59-year-old ex-smoker with GOLD Stage 3 COPD, with significant limitation to daily life and severe hyperinflation. The patient had completed pulmonary rehabilitation and was under consideration for lung volume reduction with endobronchial valves and a complementary HP gas MRI and CT clinical work-up was requested. a. HP ^3^He ventilation MRI, showing no ventilation in the right upper lobe and reduced ventilation in the left upper lobe. b. Dynamic contrast enhanced perfusion MRI, showing no perfusion in the upper lobes. C. Unenhanced CT image, indicating upper lobe-predominant bullous emphysema. D. ^3^He delayed ventilation images (three time-points during the same breath-hold), showing wash-in of MRI signal in the right upper lobe over time, consistent with collateral ventilation, which was confirmed by Chartis (gas catheter bronchoscopy). Based on these images, a decision was made to insert endobronchial valves into the left upper lobe and successful lobar collapse was seen post-procedure. After the procedure, the patient reported a significant improvement in symptoms and was able to perform the usual activities of daily living. The complementary use of multimodality imaging in the work-up for this LVRS candidate helped to prevent a likely unsuccessful right-sided procedure and provided reassurance that the left upper lobe was a viable target. COPD, chronic obstructive pulmonary disease; LVRS, lung volume reduction surgery.

**Figure 9.**
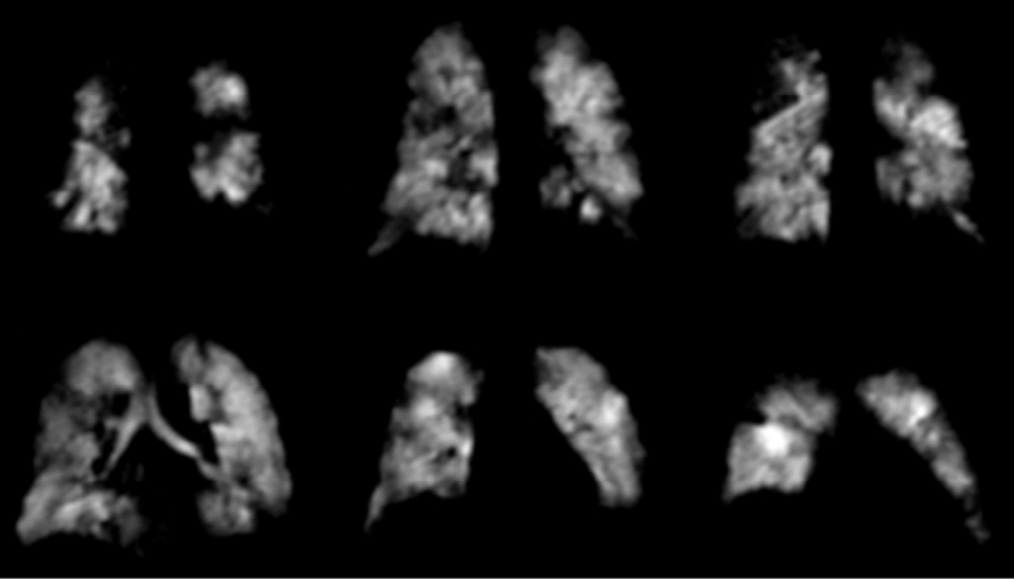
Patient G: difficult asthma in a patient with normal spirometry. Patient was diagnosed with atopic eosinophilic asthma with a high symptom burden yet normal spirometry. Medications at the time of the clinically requested MRI scan were extensive; including mepolizumab 100 mg every 4 weeks, flutiform 250/10 two puffs twice a day, prednisolone 5mgs once daily and tiotropium. Mepolizumab was seen to somewhat alleviate their asthma symptoms and reduced the number of courses of steroids resulting in a marked improvement in quality of life. However, a lower zone wheeze remained and despite long-term steroids, they still required 4—5 rescue courses of additional prednisolone in the previous 2 months. Ventilation MRI showed small and moderate defects and as a result, the decision was made to intensify patient therapy.

**Figure 10.**
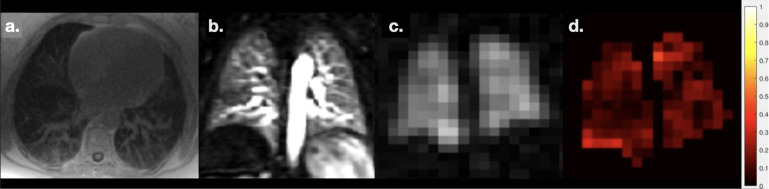
Patient H: Patient in their 60s with difficult ILD. Also diagnosed with fatty liver disease and cirrhosis. CT showed some evidence of unclassifiable ILD and gas transfer was extremely low (TL_CO_ of 26%-predicted). The patient did not meet the diagnostic criteria for hepatopulmonary syndrome and had been assessed for pulmonary hypertension. A ^1^H and HP gas MRI work-up was requested to determine the relative contribution of any ILD or PH or shunting due to hepatopulmonary syndrome, to inform a decision regarding liver transplantation. There was no evidence of shunt on dynamic contrast enhanced MRI angiography. There was mild-to-moderate ILD in the lower lobes with reticular disease seen on UTE (a), normal perfusion on DCE MRI (**b**) and no substantial ventilation defects on ^129^Xe ventilation imaging (**c**). The mean RBC/TP (whole-lung average of the map in d.) was low (0.13 compared to 0.47 in healthy volunteers), and RBC/gas was similarly low (0.0017 compared to 0.0036), while TP/gas was high (0.0134 compared to 0.0075), indicating severe interstitial thickening/endothelial diffusion-block and significant gas transfer limitation. MRI assessment supported pulmonary function gas transfer results and confirmed that gas exchange deficiency was the predominant disease process despite discordance with the apparent severity of ILD visible on CT. Key: from left-to-right: a. ^1^H UTE MRI, b. contrast-enhanced perfusion, c. Low resolution ^129^Xe ventilation imaging (obtained during a gas exchange imaging acquisition), d. Map of RBC/TP signal intensities, a metric of gas exchange. ILD, interstitial lung disease; RBC, red blood cell; TP, tissue and blood plasma; UTE, ultrashort time.

**Figure 11.**
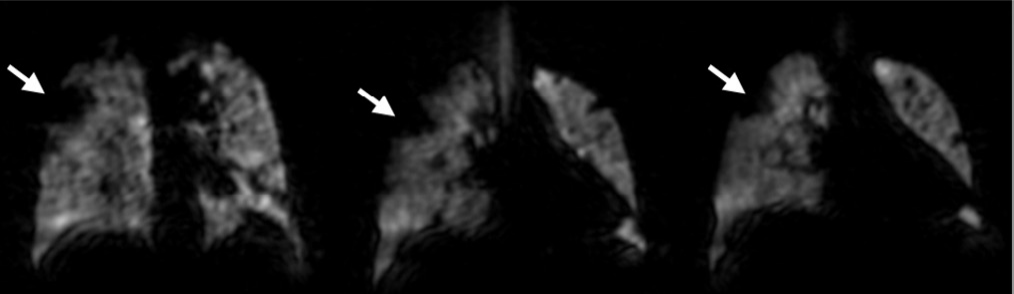
Patient I: Undifferentiated airways disease. A 71-year-old ex-smoker with progressive breathlessness on minor exertion and hypothyroidism. Spirometry was supranormal with no acute reversibility to salbutamol. No gas trapping or hyperexpansion. Normal echocardiogram. CT showed minor emphysema, disproportionate to the extent of abnormality in gas transfer (TL_CO_ 47.9%). No acute or chronic clot on CT pulmonary angiography. ^1^H perfusion MRI showed minor abnormalities. HP gas ventilation MRI showed multiple predominantly segmental ventilation defects most prominent in the right upper lobe (white arrows). MRI determined that there were substantial ventilation abnormalities despite normal spirometry, alongside relatively normal perfusion, and therefore poor local V/Q matching.

**Figure 12.**
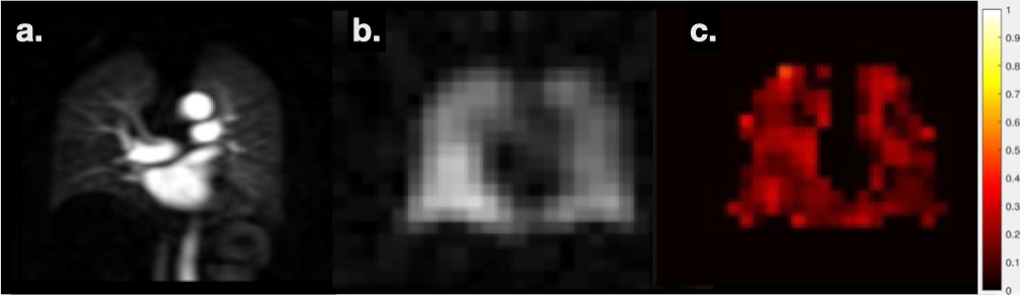
Patient J: Referred around ten months after having COVID-19. The patient had an acute infection post-COVID and persistent breathlessness; “long-COVID”. ^1^H structural and perfusion imaging revealed no apparent abnormalities (**a**). HP ^129^Xe ventilation imaging showed a homogeneous pattern of ventilation (**b**) with no substantial defects, however, significant gas transfer impairment was indicated by a mean RBC/TP (mean of map in c.) of 0.15 (c.f. healthy ~0.47) and elevated TP/Gas. Of topical note, preliminary reports of HP gas MRI in patients post-COVID-19 have been recently published^78^ and similar studies are underway at our centre to investigate long-term effects on lung function. RBC, red blood cell; TP, tissue and blood plasma.

Example clinical case studies are shown in the following figures (Figure 8, Figure 9, Figure 10, Figure 11, Figure 12).

### Future perspectives

HP gas MRI is a high sensitivity, safe and tolerable, non-ionising method for interrogation of pulmonary function, with avenues for clinical application in; (i) early (subclinical) detection, (ii) longitudinal monitoring, (iii) evaluation of treatment response. The method has great potential in drug development studies and is already established in pharmaceutical research and development pipelines. From a clinical perspective its use in characterisation of regional lung function in rare and difficult lung diseases, where CT and lung function tests have limited sensitivity and utility, are the likely areas of immediate clinical impact.

From a practical perspective, there remains a need for intervendor, inter site standardisation of HP gas MRI techniques – in particular, novel gas exchange imaging methods – that will be facilitated by multi site studies over the next few years (see: https://cpir.cchmc.org/XeMRICTC). In parallel, improvements in the availability of regulatory approved gas polarisation apparatus will allow increased accessibility of the technique in the future. Although an increasing number of multinuclear capable MR scanners are sold each year, recognition of the clinical potential of the technique by the MRI scanner vendors is still required to expedite further dissemination of this technology.
